# Values and challenges of participatory art in urban and community development: a 10-year systematic review

**DOI:** 10.3389/fsoc.2025.1571383

**Published:** 2025-09-05

**Authors:** Zichen Ke

**Affiliations:** School of the Arts, Universiti Sains Malaysia, Penang, Malaysia

**Keywords:** participatory art, urban development, community development, values, challenges

## Abstract

Participatory art is increasingly recognised as a viable intervention initiative in engaging public participation for urban and community development, effectively addressing social challenges. This study selected 20 key articles published in the past 10 years from the Web of Science (WoS) and Scopus databases, covering diverse cultural and socio-political contexts. Through thematic analysis, it identified six core values, social empowerment and democratisation, multidimensional communication, enhanced community cohesion, local cultural identity, educational promotion, and economic benefits. Additionally, it highlights the four significant challenges, including political and commercial antagonism, social participation and acceptance, sustainability issues, and resource and funding problems. The findings provide practical guidance for policymakers, practitioners, and relevant stakeholders, helping to navigate complexities, maximise the benefits of this initiative, and address the identified challenges.

## 1 Introduction

Urban and community development is a complex social project. It encompasses interconnected aspects such as public spaces, socio-cultural dynamics, political structures, economic factors, sustainability and regulating policies (Corburn, 2017; Sharifi et al., 2023). Moreover, it involves addressing the genuine needs and democratic aspirations of the populace (Held, 1992; Kempin Reuter, 2019). Reports such as the Skeffington Report (1969), Agenda 21 (1992), and the World Cities Report (2020), emphasise citizens’ voices and participatory strategies to address development issues (UK Parliament, 1969; UN, 1992; UN-HABITAT, 2020).

Furthermore, academic research has emphasised that citizens should have more say in shaping their local areas. It focuses on the different levels and forms of public participation and evaluates the limitations of these modes (Amado et al., 2010; Arnstein, 1969; Jiménez-Caldera et al., 2024). However, Eklund (1999) and Mostert (2003) point out that although theory emphasises the processes and principles that community and public participation should follow, most studies remain abstract and find difficult to investigate and verify in terms of actions and processes in actual practice. Moreover, Webler et al. (2001) note that public participation is a multi-dimensional concept and faces various challenges. For instance, factors such as the feasibility of citizen participation in local governance, legal boundaries, the appropriate timing of participation, understanding specialised terminology, sustained financial support, and sustained participation in different domains (Abas et al., 2023; Fu and Ma, 2020). Additionally, a lack of transparency in policies, bureaucratic inertia, or manipulation by external interest groups can diminish public participation and, in turn, undermine citizen confidence (Vidal and Keating, 2004; Fu and Ma, 2020; Mostert, 2003).

Given the significance of public participation in urban and community development, participatory art has become a viable and practical approach to addressing social issues through artistic means (Birchall, 2017; Jokela et al., 2015). Unlike public art and socially engaged art, participatory art can capture the participation itself and emphasise both collaboration and co-authorship between artists and participants (Bishop, 2023; Kester, 2004; Kwon, 2004). As an artistic initiative to promote social change, participatory art is increasingly recognised by organisations and the public as a vital component in driving the development processes of cities and communities (Beyes, 2010; Liinamaa, 2014; Pollock and Paddison, 2010). Focusing on the creative process within a socio-cultural framework, participatory art facilitates dialogue and communication among individuals or organisations from diverse cultural backgrounds (Li, 2024). It enhances community cohesion, promotes public engagement, and serves as a measure to drive urban development (Schuermans et al., 2012; Trienekens, 2006). According to Kester (2004), participatory art fosters understanding and social relationships among community members, facilitating consensus-building and cultural exchange. By fostering creation and interaction, it accommodates multiple cultural expressions, promoting understanding and respect across different cultural backgrounds (Bourriaud, 2002). For example, the Mural Arts Project in Philadelphia, United States, involved community members in collaborative artistic efforts to beautify their environment, address local issues, and deepen residents’ understanding of diverse cultural perspectives (Mural Arts Philadelphia, n.d.).

The participatory art process fundamentally manifests negotiation, transforming conflicts of interest into opportunities for collectively addressing social issues through artistic activities and practices (Gelfand and Dyer, 2000). Clements (2011) argues that participatory art optimistically tackles urban or community problems by enhancing the participation of diverse groups and addressing social alienation. However, such negotiated participation has also been criticised for misuse of culture and creativity as instruments of legitimation in urban processes, thereby reinforcing social structures and power relations (Park, 2016; Sacco et al., 2019). Bishop (2023) notes that participatory art, while stimulating social engagement, often lacks deep social critique or sustained impact. Its dependent and ephemeral nature might prevent it from fully realising its intentions and generating a lasting impact (Bourriaud, 2002).

Although participatory art has effectively stimulated interaction and debate in the public sphere, existing academic literature remains primarily theoretical, with limited practical evaluation. This study adopts a systematic literature review (SLR) approach to explore participatory art within urban space, community development, and civic culture, particularly its role in shaping the development of cities and communities. This article aims to provide urban planners and decision-makers with practical references for participatory art, promoting public participation in the urban sustainability process effectively, while striving to avoid excessive manipulation and instrumentalisation. The study emphasises the importance and potential value of identifying and applying participatory art in empowering communities. To support this objective, the SLR includes studies from different cultural backgrounds to capture the diversity of participatory art practices across varied socio-political and cultural settings, aiming to identify recurring values and challenges that emerge across contexts.

Based on this, the SLR synthesises peer-reviewed literature from the past 10 years and addresses the following research questions: (1) What are the recurrent values of participatory art across varied cultural and socio-political contexts in urban and community development? (2) What are the common challenges encountered in participatory art in urban and community development across these contexts?

## 2 Methods

### 2.1 PRISMA systematic review

**Figure 1:**
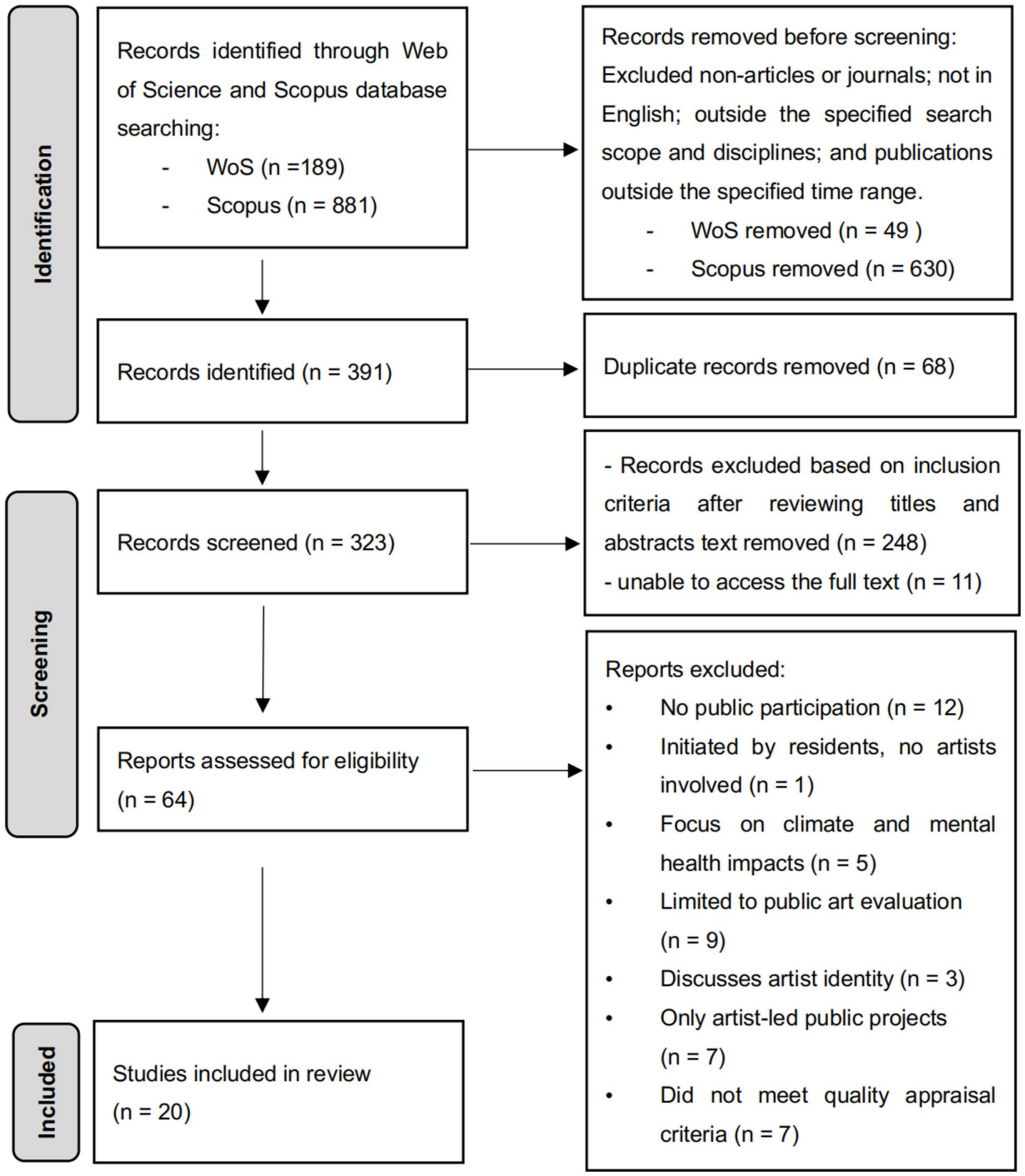
PRISMA process.

This review was conducted in strict accordance with the “Preferred Reporting Items for Systematic Reviews and Meta-Analyses” (PRISMA) guideline to ensure the integrity of this review process (Moher et al., 2010). This enhances the transparency and reproducibility of the study for other researchers in similar fields (PRISMA, 2020). Figure 1 presents the PRISMA review process.

### 2.2 Search strategies

The literature search commenced in May 2024, utilising two databases widely recognised in the field of scientific research, WoS and Scopus. This ensured that the literature included was comprehensive and of high quality (Gusenbauer and Haddaway, 2020). Table 1 details the inclusion and exclusion criteria for this SLR. The study focused on literature published in the past decide to analyse the latest research developments and trends within the field.

**Table 1:** Selection criteria.

Category	Inclusion	Exclusion
Literature Type	Peer-reviewed journal articles.	Non-peer-reviewed articles, literature reviews, monographs, conference proceedings, book reviews, reports, and grey literature.
Language	Full-text articles in English	Articles with English abstracts but non-English full texts; Articles with only English titles but full content in other languages.
Accessibility	Articles with accessible abstracts and full texts.	Articles are unavailable electronically or through other means.
Coverage	Literature on participatory art within urban or community contexts; Research on participatory art projects.	Theoretical studies without practical focus; Literature on participatory art outside urban contexts.
Community/ Resident Participation	Literature on community/resident participation.	Articles on art projects implemented solely by artists/governments; Literature without community/resident participation.
Publication Date	Publications from 2013 to April 2024.	Publications outside 2013–April 2024.

#### 2.2.1 Literature search and identification

In this study, the identification focused on the terms of “participatory art” and “urban development.” It involves identifying a series of keywords, their synonyms, and related phrases. First, the keywords associated with “participatory art” highlight art forms that involve direct public participation. This approach emphasises community engagement and social interaction throughout the creation process rather than concentrating solely on the artwork itself. In the context of urban cross-cultural negotiation, these terms highlight how participatory art, as a form of social practice, promotes dialogue and understanding among different cultural groups through artistic activities. This enhances community cohesion and builds consensus in a multicultural urban environment.

In the context of “urban development,” the keywords selected encompass a wide range of topics related to urban planning and development, including policies, social and cultural dynamics, and public spaces. It focuses on addressing the challenges brought by urbanisation and explores how art can be used to promote social inclusiveness and cultural diversity in urban spaces. Additionally, literature search also encompassed keywords related to community-level participation and collaboration. Those are relevant and crucial for understanding how participatory art can mobilise and inspire community actions or involvements. This section highlights community-driven projects that foster cross-cultural dialogue and collaboration, linking participatory art to urban development strategies and practices. Table 2 displays the search by terms and concepts, specifying the relevant key terms, keywords and related phrases for use in the search. During the retrieval process, Boolean operators (AND, OR, NOT) are used to optimise the search combinations and allow for variations of the search terms, further expanding the search coverage, capturing more potentially relevant literature, and ensuring the accuracy and comprehensiveness of the search results.

**Table 2:** Search terms for the SLR.

Search concepts	Definitions	Keywords & related phrases
Participatory art	Participatory art is an artistic practice where the public collaborates with artists to strengthen community involvement and social interaction. It emphasises the experience of participants, interactions, and transformation within the community during the creation process (Bishop, 2023; Helguera, 2011; Kester, 2004).	Participatory art, socially engaged art, community art, public art, collaborative art, artistic collaboration, artistic intervention
Urban development	Urban development typically refers to planning and constructing urban public spaces, implementing related policies and cultural activities, and resolving social services and environmental issues (Batty, 2008; Yigitcanlar and Teriman, 2015).	Urban development, urban planning, urban regeneration, city planning, urban growth, urbanisation, urban policy, urban social development, urban cultural, urban resilience, public spaces, city revitalization, urban life, urban design, place making, spatial development, community engagement, community collaboration, community building, community development, community governance, community action

Specifically, the search strategy was adjusted according to the retrieval formats of two databases. In WoS, searches were executed using the TOPIC field; while in Scopus, the TITLE-ABS-KEY field was utilised. The query string included the following terms related to participatory art and urban studies: (“participatory art*” OR “socially* engaged art*” OR “community art*” OR “public art*” OR “collaborative art*” OR “artistic* collaboration*” OR “artistic* intervention*”) AND (“urban development” OR “urban planning” OR “urban regeneration” OR “city planning” OR “urban growth” OR urbanisation OR “urban policy” OR “urban social development” OR “urban cultural*” OR “urban resilience” OR “public spaces” OR “city revitalisation” OR “urban life” OR “urban design” OR “place-making” OR “spatial development” OR “community* engagement” OR “community collaboration” OR “community building” OR “community development” OR “community governance” OR “community action”).

In WoS, identified articles were limited to those indexed in the Science Citation Index Expanded (SCI-Expanded), Social Sciences Citation Index (SSCI), and Arts & Humanities Citation Index (A&HCI). In Scopus, searches were restricted to disciplines within the Social Sciences and Arts and Humanities. Non-article or non-journal publications and articles not written in English were excluded. It is worth noting that the search strategy adopted in this review prioritised conceptual and thematic relevance over geographic representation. This decision reflects the current state of the field, in which case-based empirical research remains relatively limited and theoretical discussions are more prevalent. Based on the above search strategy and exclusion criteria, 391 publications were selected for further screening.

The retrieved articles were organised into an Excel spreadsheet and categorised by title, filtering out 68 duplicates from the two databases. The remaining 323 articles were manually screened by reviewing their titles and abstracts against the established criteria. During this process, 248 articles unrelated to urban domains, public participation, and participatory art practices, along with 11 publications lacking full-text access, were excluded. Ultimately, 64 publications were retained for quality assessment, with the screening process completed on 19th June 2024.

Additionally, in the literature, the terms “participatory art,” “public art,” and “socially engaged art” are sometimes used interchangeably. In order to ensure a comprehensive search, these terms were included in the search string. However, final inclusion was restricted to studies meeting the definition of participatory art, which refers to involving co-creation/collaboration between artists and participants (Bishop, 2023; Helguera, 2011; Kester, 2004). This was applied during the purposive sampling phase described in Section 2.2.2.

#### 2.2.2 Quality assessment of the selected articles

The literature assessment stage involved reading the full text of each study to ensure research quality, reduce information bias, and maintain validity. This process followed the SLR standards, applying consistent inclusion and exclusion criteria to minimise bias and ensure that the selected studies met the core requirements of this review. Following the initial screening, 64 articles proceeded to the quality appraisal stage. Firstly, a purposive screening strategy was applied to identify the type and content of each study, based on three key inclusion criteria: (1) whether the study contributed to understanding the role of participatory art in urban and community development; (2) whether the study provided clear insights; and (3) whether the study met the definitional criterion of participatory art. Based on this step, 32 articles were excluded.

Secondly, the remaining studies were assessed using a structured quality appraisal process. While standardised quality appraisal tools exist, they do not fully capture the specific features of participatory art aimed at promoting urban and community development. As noted by Batten and Brackett (2021), when no single tool is fully applicable, it is possible to develop or adapt existing checklists or tools to suit the specific research context. Based on this, relevant checklists from the Joanna Briggs Institute (JBI, 2020) and the Critical Appraisal Skills Programme (CASP, 2023) were selected. These were then adapted to produce the final checklist: (i) relevance to the specific research focus; (ii) provision of practical research insights into participatory art in urban or community contexts; (iii) alignment between methodology and research objectives; (iv) rigour of data or case analysis; (v) adequacy of evidence supporting the conclusions; and (vi) contribution to the field. Before full application, the checklist underwent a brief pilot test and minor refinements to ensure clarity, relevance, and consistency in interpretation. Each item was scored on a three-point scale (low = 0; medium = 1; high = 2), giving a total possible score of 0–12. A score below 6 (less than 50% of the total score) indicated insufficient quality across multiple key criteria and resulted in exclusion.

An external researcher specialising in participatory art and creative city studies was invited to independently verify the quality assessment results. This researcher was not involved in the study and contributed solely during the verification stage. This arrangement was intended to minimise potential bias and subjectivity, thereby enhancing the reliability of the assessment process. Any differences were resolved through discussion until consensus was reached. In total, 20 articles were included in the analysis (Table 3). The final selection stage was completed on 21^st^ August 2024.

**Table 3:** Review of selected publications.

Author (year)	Region	Focus	Organiser/Initiator
Sacco et al. (2019)	USA	Urban renewal & power relations	Artist & Non-profit organisation
Fobear (2017)	Canada	Community fringe groups (LGBT) & Sustainable development of communities	NGO
Tan (2019)	Singapore	Public space, cultural policy, urban planning & urban transformation	Government; Artist
Lavrinec (2014)	Lithuania	Neighbourhood redevelopment & community revitalisation	Communities & residents
Lee (2013)	USA	Community Development for Multicultural & Ethnic Groups	Non-profit organisation
Uskoković (2017)	Croatia	Local architectural heritage conservation & sustainable community development	Artist
Kelaher et al. (2014)	Australia	Community development & community well-being	Government institution
Grant-Smith and Matthews (2015)	Ireland	Urban culture & community development	Non-profit organisation
Kortbek (2019)	Denmark	Placemaking	Government & cultural institution
Guzzo (2021)	Brazil	Reshaping urban space, interaction & dialogue within communities	Government institution
Hanser (2020)	UK	Community dialogue & relationship building	Artist
Li and Pang (2022)	Belgium	Revitalization of urban spaces	Non-profit organisation
Phillips and Tossa (2017)	Thailand	Intergenerational (children & elderly) and intercultural exchanges in the community	Non-profit organisation
Eynaud et al. (2018)	France	Reshaping urban spaces & empowering communities	NGO & CSO
Sachs Olsen (2018)	Switzerland; UK	Personal experience and collective memory of urban space; Imaginations and visions of cities; Marginal groups.	Non-profit organisation
Smith (2022)	USA	Community interfaith culture & sustainability	Non-profit organisation
Crisman (2022)	USA	Equity & inclusion in urban communities	Community
Wiberg (2022)	Sweden	Urban spatial planning & sustainable development	Government
Haedicke (2016)	France	Community conflict & social integration	Government
Irwandi et al. (2023)	Indonesia	Improve the backwardness of urban villages	NGO & CSO

#### 2.2.3 Data abstraction and analysis

This study conducts thematic analysis to identify and organise themes closely related to the research question. According to Braun and Clarke (2006), the analysis began with an in-depth examination of the data, followed by identifying initial codes which were systematically organised into potential themes. A detailed review ensured these themes accurately reflected the core content of the literature. Ultimately, these themes were clearly defined, named and structured into a thematic report corresponding to the research questions. Given the manageable number of sources and the thematic nature of this review, coding was conducted manually and organised using Excel spreadsheets. The analysis developed 10 themes: six potential advantages of participatory art and four key challenges.

## 3 Results

### 3.1 Overview of selected literature

**Figure 2:**
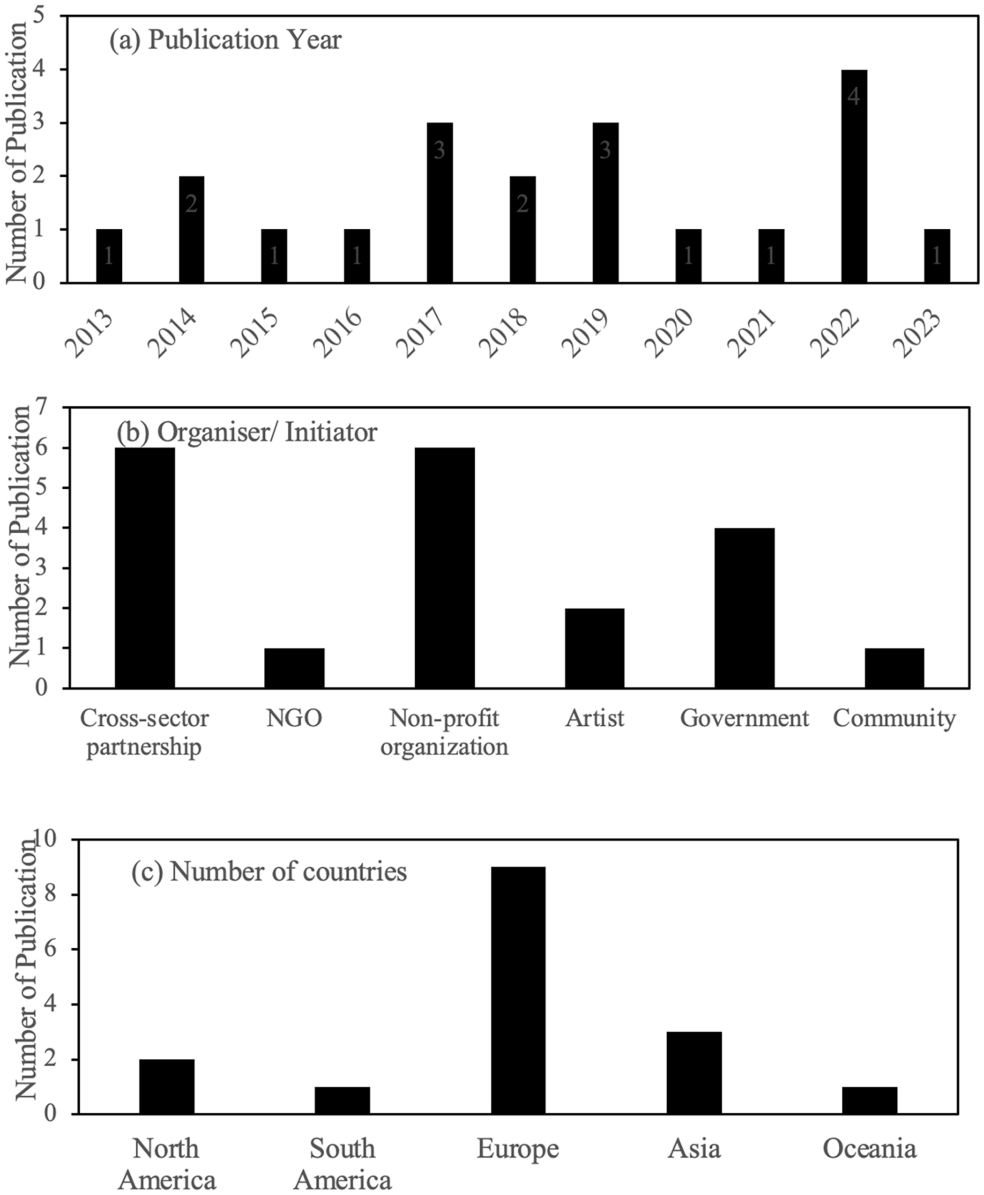
Publications on participatory art: **(a)** Number of publications by year; **(b)** Types of organisers/initiators leading participatory art projects; **(c)** Number of publications from countries grouped by region.

The 20 articles assessed in this study originated from 17 countries, spanning developed and developing nations across diverse economic contexts and political systems. Europe has the highest coverage, including nine countries such as Lithuania, Croatia, Ireland, and others, as shown in Figure 2c Southeast Asia follows with three countries and North America with two countries. Besides, Brazil and Australia have also contributed to this field. Figure 2a illustrates the temporal distribution of publications, with the highest number of articles (four) published in Crisman, 2022. Figure 2b displays data on the research organisations and initiators. Mostly, research on participatory art was typically led by cross-sector collaborations and nonprofit organisations. They are followed by government organisations and agencies, and next by individual artists. In comparison, there are fewer participatory art projects initiated solely by NGOs or community groups. Additionally, Table 4 summarises the characteristics of these articles, including participatory art approach, duration, main findings, and limitations.

**Table 4:** Characteristics of the included studies: participatory art approach, duration, main findings, and limitations.

Author (year)	Participatory art approach	Duration	Findings	Limitations
Sacco et al. (2019)	Temporary art & community activity platforms; situational performances & street actions	6 months; short-term project	Easily incorporated into top-down planning; can facilitate the reactivation of urban rights; helps build trust & enhance community assets & capacities	Lack of long-term follow-up evaluation; susceptible to political & institutional power structures
Fobear (2017)	Participatory community mural	5 consecutive Saturdays	Enabled participants to lead the narrative, intertwining personal messages with direct political demands, and fostering social empowerment and democratisation	Short duration, limited sustainability of impact
Tan (2019)	Participatory art interventions in multiple contexts & settings (dialogical live installations & performances, public collaborative installation interactions, community installations)	Several months	Stimulated public participation; educational significance; redefined spatial & cultural meanings	Short duration, limited sustainability of impact; constrained by national & institutional frameworks
Lavrinec (2014)	Street co-creation (public space beautification activities)	Several months	Fostered community mutual aid & trust networks; promoted informal learning & co-building of skills	Depth and coverage of participation limited by time; required communication & collaboration with government
Lee (2013)	Community mural & sculpture creation, art workshops	Several months	Promoted communication & understanding among diverse cultural groups; enhanced community cohesion & identity	Limited duration, uncertain long-term impact
Uskoković (2017)	Community art actions (community choreography, artist residencies, participatory theatre)	Several years	Strengthened residents’ cohesion & rebuilt community trust; educational tool; facilitated funding for urban architectural conservation projects; build cultural identity	Internal community interest conflicts & lack of consensus for unified action; influenced by political &economic factors
Kelaher et al. (2014)	Visual arts, theatre, performance	3 years	Increased community awareness & discussion of social issues such as mental health, discrimination, & violence; strengthened community resonance & action	Conflicts between artistic goals & funding body criteria
Grant-Smith and Matthews (2015)	Mural	Several weeks	Enhanced community cohesion & civic identity; reinforced cultural memory & intergenerational exchange; enhance economic potential	Political & cultural conflicts; low initial public acceptance; used for commercial purposes
Kortbek (2019)	Installation art; community co-building & renovation; collective storytelling of the city	Several months	Stimulated collective creativity & promoted social inclusion; generated diverse social & cultural interactions in different communities	Mismatched expectations between artists & residents; time constraints limited depth of collaboration; differences in approach & value orientation from government cultural policy vision; commercially instrumentalised
Guzzo (2021)	Performance intervention	4 months	Established long-term relationships with the community in marginalised urban areas through artist residencies; created a “shared space” that fostered cross-group and cross-regional collaboration networks	Risk of being appropriated & institutionalised by political or cultural institutions; reliance on external support & resources limits sustainability
Hanser (2020)	Cross-street outreach / outdoor education / community art	Multi-stage	Fostered connection	Reliance on multiple channels & external support limits sustainability
Li and Pang (2022)	Multimedia and cross-media participatory art (photography, publications, exhibitions)	3 years	Transformed urban voids into inclusive public spaces; enhanced community cohesion; resisted erosion of community culture	Risk of instrumentalization; reliance on external resources; vulnerable to policy changes and other external threats; participation may be limited
Phillips and Tossa (2017)	Action and narrative (walking as participatory art, storytelling & children’s curation)	Multi-stage	Intergenerational & intercultural learning; community communication & shared storytelling	Sustainability challenges; limited community public acceptance; reliance on external funding
Eynaud et al. (2018)	Spatial co-transformation	Multi-stage	Reconstructed urban rights; strengthened community identity & social bonds; facilitated negotiation among residents, institutions, and government policies; promoted resource acquisition	Institutional resistance from government; reliance on funding; challenges in sustaining resident participation and engagement
Sachs Olsen (2018)	Community co-creation (collecting objects, sounds, images, workshops)	Short-term	Created open spaces to encourage expression of diverse voices and foster a sense of belonging and participation	Overreliance on institutional frameworks & constraints set by organisations
Smith (2022)	Exhibitions, forums, dialogues, shared meals	Multi-stage	Built relationships and promoted dialogue; activated community networks & social inclusion; challenged prejudices	Politicisation concerns; limited public/resident participation & acceptance
Crisman (2022)	Artistic co-creation / community organising (workshops, cultural festivals, etc.)	Multi-stage	Bottom-up co-creation approach; prompted government/institutional response to grassroots co-creation demands; amplified community voices & influenced public land development; community identity & sense of belonging	Resident participation remained largely symbolic; issues of social participation & acceptance; sustainability & funding challenges
Wiberg (2022)	Artist residencies & community co-creation	2 years	Fostered democratic participation & knowledge production; triggered spatial and institutional changes; promote participation & collaboration	Art instrumentalised & politicised; limitations in resolving problems
Haedicke (2016)	Community co-creation art projects, collaborative installation performances, and temporary art projects	20 months	Transformed a subway station from a symbol of violence into a space for cooperation; alleviated community tensions through indirect contact (shared art creation) and fostered collaboration; shifted residents from passive observers to active participants, spurring subsequent community art projects; enhance dialogue and community cohesion	Lack of long-term mechanisms to sustain & resolve conflicts, and issues with sustaining residents’ participation; phased funding & continued reliance on public support
Irwandi et al. (2023)	Community murals, festivals	Multi-stage	Reset local narratives, fostered sense of belonging, mobilised residents and multi-stakeholder collaboration, boosted tourism & environmental improvement	Funding dependence; low resident participation; insufficient long-term sustainability; A tool for commercial tourism

### 3.2 The potential values of participatory art

This study identified six emerging themes of potential values in participatory art from the 20 selected articles. The themes cover social empowerment and democratisation, multidimensional communication, enhanced community cohesion, local cultural identity, educational promotion, and economic benefits.

#### 3.2.1 Social empowerment and democratisation

a  Authentic interaction.

Fair and transparent decision-making is a key factor in driving local policy changes. In this context, participatory art in public spaces underscores the necessity of genuine interaction with community members. Evidence from four studies (Grant-Smith and Matthews, 2015; Sacco et al., 2019; Guzzo, 2021; Sachs Olsen, 2018) highlights how legitimised art projects involve and engage local residents, transforming them from passive executors to active co-creators. This role transformation further encourages community members to participate in the revitalization of public spaces, urban planning and development, and environmental discussions.

b  Democratic participation.

Artistic creation enhances the sense of democratic participation within the community. Community members are encouraged to express their opinions freely and participate actively in open dialogues, which will enhance their critical thinking and self-reflection. Findings from five studies (Grant-Smith and Matthews, 2015; Fobear, 2017; Crisman, 2022; Wiberg, 2022; Sachs Olsen, 2018) show that community members are encouraged to express their opinions freely and participate actively in open dialogues, enhancing critical thinking and self-reflection. As emphasised by Grant-Smith and Matthews (2015) and Fobear (2017), listening to the voices of marginalised groups is crucial for promoting inclusivity and diversity within the community. Participatory art provides a platform for residents from different backgrounds to engage in meaningful communication and interaction, bridging traditional social barriers and fostering community integration.

Participatory art has greatly cultivated residents’ interest and involvement in local governance. It enables the government and policymakers to listen to and address the community’s needs, particularly in areas with low social participation. This initiative strengthens community engagement and feedback mechanisms in the policy-making process (Crisman, 2022; Wiberg, 2022), fostering trust and a sense of responsibility towards local government among local community. It encourages them to actively monitor and participate in the implementation of local policies, promoting transparency and fairness in governance. This approach facilitates greater community engagement, ensuring that policy-making reflects not merely the official perspectives but also takes into consideration of specific needs and expectations of a wider resident population.

#### 3.2.2 Multidimensional communication

a  Cross-cultural and cross-group communication.

Evidence from six studies (Guzzo, 2021; Kelaher et al., 2014; Fobear, 2017; Lee, 2013; Sacco et al., 2019; Sachs Olsen, 2018) shows that participatory art plays a crucial role in building a more inclusive and harmonious social environment by redefining and revitalising public spaces as central venues for community engagement and cultural exchanges. This process enhances the social function and cultural value of public spaces by breaking down cultural barriers and language obstacles and facilitating cross-cultural exchange and collaboration among all parties. It enables individuals to easily express experiences that are often difficult to articulate through language (Guzzo, 2021). Artistic expression significantly enhances urban residents’ understanding of each other’s cultures (Kelaher et al., 2014). This helps foster tolerance and acceptance of diversity among different ethnicities, groups, and communities in addressing the challenges faced within the community and promoting sustainable urban development. This initiative sparks public discussions and collaborative efforts, effectively diminishing social divides and cultural barriers while enhancing cooperation among people from diverse backgrounds (Fobear, 2017; Lee, 2013; Sacco et al., 2019; Sachs Olsen, 2018).

b  Intergenerational, cross-disciplinary, and cross-class communication.

Findings from three studies (Smith, 2022; Phillips and Tossa, 2017; Crisman, 2022) highlight how participatory art fosters mutual understanding across generations, disciplines, and social classes. Participants work together to build a harmonious and inclusive community environment by promoting understanding and respect through art forms (Smith, 2022). As a medium, participatory art greatly contributes to mutual understanding among community members. Phillips and Tossa (2017) highlight that art activities not only link people of different ages and cultural backgrounds to communicate but also enable learning through interactions between children and adults, which further strengthens the inclusiveness and diversity of the community. Crisman (2022) further emphasises that participatory art tends to diminish the dominant role of government agencies and big business in the traditional planning process. However, it underscores the active participation of all community members in the planning process. Through cross-class and interdisciplinary communication, people of different socioeconomic statuses and professional backgrounds come together, breaking down power hierarchies in urban planning and knowledge generation, bridging social stratification gaps, and forming a collaborative bottom-up model of creation. This model promotes the sharing of knowledge and experience across fields (Crisman, 2022). This diverse participatory approach enables participants to understand social and cultural issues holistically, fostering horizontal collaboration among multiple stakeholders within the community and enhancing interaction and mutual understanding between community members and government officials, thereby jointly addressing and solving urban and community challenges (Crisman, 2022).

#### 3.2.3 Enhanced community cohesion

a  Shared spaces.

Evidence from five studies (Fobear, 2017; Li and Pang, 2022; Phillips and Tossa, 2017; Lavrinec, 2014; Hanser, 2020) shows that art projects, as shared spaces for experience and creation, encourage community members to connect and collectively express themselves, fostering interaction and understanding. As noted by Fobear (2017), Li and Pang (2022), Phillips and Tossa (2017), and Lavrinec (2014), the construction of these shared experiences significantly enhances community cohesion, allowing members to co-create and share emotions and experiences through artistic activities. Participatory art creates an informal social setting that offers opportunities for open sharing and spontaneous civic commonality. As emphasised by Hanser (2020), participatory art helps to bridge the gap between social service providers and community members, building trust and cooperative relationships through shared participation and dialogue in art.

b  Diversity in participation and expression.

Findings from seven studies (Eynaud et al., 2018; Wiberg, 2022; Crisman, 2022; Haedicke, 2016; Irwandi et al., 2023; Kelaher et al., 2014; Smith, 2022) indicate that inviting community members to participate directly in artistic creation enhances their sense of ownership and involvement in their living environment. Studies by Eynaud et al. (2018) and Wiberg (2022) show that this participation has expanded through participatory art into the planning and decision-making processes, promoting broader community participation and collaboration. According to Crisman (2022), the involvement of grassroots actors in art and cultural activities—particularly in multicultural and multiethnic settings—can influence urban planning and community development while fostering a sense of community identity, cohesion, and belonging. Haedicke (2016) further explains that public participation in participatory art activities such as street performances and public art installations encourages cooperation and understanding among various community members, thereby strengthening community ties. Irwandi et al. (2023) emphasise that engaging in artistic activities to express and preserve local cultural heritage and stories effectively promotes community well-being and maintains shared local memories among the community. This approach not only fosters unity and participation within the community but also enhances overall community cohesion (Kelaher et al., 2014; Smith, 2022).

#### 3.2.4 Local cultural identity

a  Collective memory and cultural transmission.

Insights from four studies (Tan, 2019; Irwandi et al., 2023; Uskoković, 2017; Grant-Smith and Matthews, 2015) show that participatory art not merely explores the resonance or connections between collective memory and personal histories but also involves collective review and investigation of the cultural significance of a specific location or site. This practice encourages participants to develop an understanding of collaborative collectivism and self-organisation. It serves as a form of social action and a deep cognitive process to enhance community members’ reflections on local culture through artistic activities, thereby strengthening community and public identity and local pride (Irwandi et al., 2023). Uskoković (2017) acknowledged the role of urban buildings as focal points link local cultural sentiments and cultural activities. Participatory art can significantly enhance residents’ awareness of the importance of architectural heritage and their sense of identity. This aligns with Grant-Smith and Matthews’s (2015) argument that artistic creations reflect the history and cultural narratives of the community. Such engagement facilitates a better understanding and appreciation of their shared history and cultural heritage among local communities and the general public, thus promoting their protection of local culture and its effective inheritance.

b  Revitalising public spaces.

This view is supported by evidence from three studies (Guzzo, 2021; Li and Pang, 2022; Sachs Olsen, 2018). Guzzo (2021) highlights that participatory art is a valuable tool for reshaping and revitalising spaces. It helps to enrich public spaces’ social and cultural functions by transforming them into central venues for community activities and cultural exchange. These public spaces do more than display art. They become platforms that encourage social and community interaction, co-creation, cultural development, and effective communication (Sachs Olsen, 2018). Artistic interventions transform these spaces into inclusive and multifunctional community resources that positively influence the city’s cultural ecology. These activities are comprehensive and inclusive, engaging diverse communities and groups, including marginalised populations, low-income individuals, ethnic minorities, and people from various backgrounds and professions. This broad participation enhances the cultural significance and function of public spaces, promoting diversity, cultural integration, and exchange between different communities (Li and Pang, 2022).

#### 3.2.5 Educational promotion

a  Broad social education.

Participatory art demonstrates its significant role in subtly shaping public education and fostering social interaction through artistic interventions in public spaces. Three studies support this potential (Tan, 2019; Uskoković, 2017; Phillips and Tossa, 2017). Tan (2019) points out in his research that through strategies of socialisation, dialogue, and collaboration, art projects not only showcase the social commentary function of artworks but, more importantly, emphasise the educational significance of the creation process and interaction with the audience. These projects, such as paintings, installations, and performance art, aim to inspire public awareness and reflection on social changes around them, highlighting the potential of participatory art in promoting social engagement and public education.

In terms of urban renewal and architectural preservation, Uskoković (2017) believes that creative artistic interventions in social practices promote education and community building. Open activities and discussions not only allow residents to directly participate in the preservation of buildings and community regeneration but also effectively transform artistic practices into educational tools that enhance residents’ awareness and sense of responsibility towards their living environment, promoting learning and exchange within the community.

Phillips and Tossa (2017) highlight the educational value of participatory art in fostering community interaction and intergenerational exchange. They point out that this bidirectional learning process, particularly the interaction between children and adults, enhances the social skills and independence of both parties and also broadens participants’ opportunities for education and proactive learning, improving creative thinking and problem-solving abilities. Through artistic interventions, participants are encouraged to think from new perspectives within a broad community and cultural environment, deepening their understanding of social participation and cultural issues.

b  Knowledge sharing and skills education.

Lavrinec (2014) explores how art projects have become platforms for knowledge sharing and skills transfer among community residents. In informal, interactive environments, residents learn from each other and impart various skills, such as making ceramic mosaics, understanding the value of public art, and developing interaction and communication skills within the community. These activities positively impact the community’s long-term development and significantly promote personal growth and development. Through hands-on practice, participants develop their design perception and aesthetic abilities and strengthen internal community bonds. This helps to boost self-efficacy and a sense of responsibility, enabling them to play a more active role in future planning and decision-making initiatives (Lavrinec, 2014). Furthermore, from a macro perspective, whether during the initial recruitment phase or throughout the implementation process of participatory art, both internal participants, external observers, and indirect activity contacts can become conduits for knowledge transmission, thereby achieving widespread dissemination of information and skills.

#### 3.2.6 Economic benefits

In the reviewed literature, the economic benefits of participatory art were not a primary focus, yet two studies reported this finding (Grant-Smith and Matthews, 2015; Irwandi et al., 2023).

a  Tourism and commerce.

Grant-Smith and Matthews (2015) indicate that beautifying the urban environment enhances the visual appeal of the community spaces, directly boosting tourism potential. Participatory art can attract more visitors, thereby increasing tourism revenue and indirectly promoting the development of local businesses such as dining, retail, and service industries. Furthermore, this collaborative approach strengthens connections among community members. It increases their willingness to invest in and maintain their community spaces, indirectly revitalising the local economy, creating job opportunities, and contributing to economic diversification.

b  Local economic growth.

The study by Irwandi et al. (2023) focuses on shaping local identity through participatory art. These art activities effectively transformed former slums into tourist destinations, significantly enhancing the area’s visibility and attractiveness, profoundly impacting local economic development, and driving local economic growth. In this context, it can be inferred that such economic development undoubtedly encourages further government investment and support, stimulates additional private and international capital investment, and accelerates improvements in infrastructure and services. This further solidifies the area’s status as a vibrant tourism and commercial hub. Grant-Smith and Matthews (2015) suggest that this initiative unlocks economic potential, providing new opportunities for local economic diversification and sustainable development.

### 3.3 Challenges of participatory art

Despite the significant potential values of participatory art in the six aspects mentioned above, it faces several challenges and criticisms. These challenges often originate from the execution intentions of the initiators and designers, as well as the complex interactions with community dynamics. This study identified four thematic challenges: political and commercial antagonism, issues of social participation and acceptance, sustainability issues, and resource and funding problems.

#### 3.3.1 Political and commercial antagonism

a  Politicisation tool.

Findings from three studies (Guzzo, 2021; Sachs Olsen, 2018; Smith, 2022) indicate that, in social and political contexts, participatory art can be a double-edged sword, with both positive impacts and potential risks. Guzzo (2021) notes that combining art and politics is always challenging. While this fusion can elevate art as a medium for social and political change, it can also turn it to a mere political instrument. Within a culture-driven environment, artistic practices may become mechanisms of supremacy or elite-driven strategy. Although they seem to promote public participation and empower communities, these initiatives can actually function as top-down control mechanisms. They may legitimise the transformation of urban spaces for hidden agendas through artistic practices (Sacco et al., 2019). This politicisation can restrict artistic freedom, turning art activities into tools that serve specific political agendas or policy objectives (Smith, 2022).

b  Commercialisation tool.

Evidence from four studies supports the view that commercialisation can undermine the social objectives of participatory art (Grant-Smith and Matthews, 2015; Irwandi et al., 2023; Kortbek, 2019; Sachs Olsen, 2018). Although participatory art aims to be non-commercial, addressing urban and community-related issues, successful art activities often attract commercial attention, which can lead to changes in its original community structure and nature, resulting in the rapid development of local dining, retail, and service industries (Grant-Smith and Matthews, 2015; Irwandi et al., 2023). According to Kortbek (2019), artists and curators often intentionally control the outcomes of their projects. This can involve using art to deflect social criticism in pursuit of commercial objectives (Sachs Olsen, 2018). Such commercialisation trends can divert participatory art from its original intent, potentially resulting in exclusion and division within the community. This raises concerns about the social value and sustainability of these artistic efforts.

c  Antagonism and conflict.

Findings from two studies (Wiberg, 2022; Haedicke, 2016) highlight this issue. When art projects become tools for policy or commerce, they may cause conflicts or unfriendly relationships, particularly among different stakeholders (Wiberg, 2022). Different expectations on goals, needs, and resource allocation expectations can lead to tension and conflict. Although art and cultural activities aim to promote community integration through inclusivity and participation, in practice, divergent voices and conflicting interests can result in antagonism and inharmoniousness. Such conflicts of interest may hinder the full respect of the community’s needs and desires, thereby blurring the boundaries of participatory art and limiting its independence and creativity (Haedicke, 2016).

#### 3.3.2 Issues of social participation and acceptance

a  Community and public acceptance.

Findings from four studies (Kortbek, 2019; Eynaud et al., 2018; Haedicke, 2016; Smith, 2022) highlight that gaining community and public acceptance remains a primary challenge for participatory art. Kortbek (2019) argues that while some participatory art projects aim to promote community inclusivity and beautify the environment, their effectiveness often diverges from policy goals. These projects may lead to dissatisfaction and resistance when they do not meet community expectations. For instance, artists’ political or commercial motives may become apparent, which might not align with the true needs of community members. This gap of understanding exists not only within specific communities but also within the broader framework of cultural policy. To ensure the success of participatory art, broad public participation and consensus-building are crucial and essential. Artists and organisers must deeply understand and address the specific needs and expectations of the community.

Additionally, Eynaud et al. (2018) point out that this art form may have structural vulnerabilities, as it relies on extensive social networks and long-term project planning to achieve significant impact. Haedicke (2016) argues that although art projects aim to alleviate internal community tensions through collaborative creation and participation, some groups remain sceptical of these initiatives, worrying they may disrupt the existing social order (Smith, 2022). This suggests that a primary challenge for participatory art projects is to gain broad community support and acceptance, particularly in situations with deep-seated divisions and opposition within the community.

b  Acceptance at the level of popular culture.

Findings from two studies (Irwandi et al., 2023; Li and Pang, 2022) highlight that within the mainstream cultural system, participatory art often faces challenges in gaining acceptance due to community and societal factors. Irwandi et al. (2023) highlight that while these art projects aim to foster creativity and encourage economic development, they can face rejection due to the unique needs of each community. This is especially true in areas with complex socio-economic conditions or high cultural diversity, where art activities may be viewed as too high-end or not aligned with local culture (Li and Pang, 2022). As a result, some communities may feel alienated and struggle to recognise the value of these initiatives.

Li and Pang (2022) further observed that even though participatory art has significantly enhanced community participation and cultural life, its social acceptance during implementation remains challenging. The diverse level of cultural understanding makes specific art projects difficult to understand and hard to promote among public communities. Governments and the public often prioritise commercial interests or security issues, overlooking the long-term contribution of art to community building. This discrepancy causes community members to feel that these art activities do not reflect their lives and cultural identities (Li and Pang, 2022). The view of these art activities as elite culture makes individuals feel they lack a voice in community decision-making.

#### 3.3.3 Sustainability issues

a  Form and structural limitations.

Findings from two studies (Kortbek, 2019; Wiberg, 2022) highlight that although participatory art can promote interaction among community members, to maintain its structural sustainability is a great challenge. The primary issue is the general lack of awareness and understanding of the profound impacts of cultural strategies. Often, these art projects are viewed as temporary and situational initiatives. Some people see them as merely superficial enhancements to community spaces, rather than as a tool capable of addressing broader social issues (Kortbek, 2019). While participatory art can increase engagement locally, it may seem insufficient in tackling widespread societal structural problems (Wiberg, 2022).

b  Limitations of impact.

Evidence from five studies (Phillips and Tossa, 2017; Crisman Crisman, 2022; Haedicke, 2016; Li and Pang, 2022; Sachs Olsen, 2018) indicates that participatory art projects can significantly impact urban policy and development. However, their long-term effects and adaptability to social and environmental changes face major challenges. Phillips and Tossa (2017) stress the consideration of investment to perform ongoing assessment or evaluation to ensure the long-term impact of these projects. Crisman (2022) highlights that while these art projects have a significant short-term impact, their long-term sustainability is still uncertain. Haedicke (2016) adds that predicting and measuring these impacts in relation to social structure and cultural attitudes is challenging. In the pursuit of social outcomes, the artistic quality of the works may be overlooked by project organisers or participants in these activities, which could limit the overall impact and sustainability of the projects. As a result, community engagement may diminish after the project’s conclusion, making it difficult to maintain momentum and achieve lasting effects (Li and Pang, 2022; Sachs Olsen, 2018).

c  Dependency limitations.

Findings from three studies (Kortbek, 2019; Li and Pang, 2022; Eynaud et al., 2018) show that the dependency of participatory art projects is significantly limiting their sustainability. Firstly, these projects often require a thorough understanding of local cultural policies and social dynamics. Some projects fail to achieve their intended goals due to a lack of this understanding, which can intensify internal divisions and conflicts (Kortbek, 2019). Second, the success of these initiatives hinges on the active participation of both artists and community members. If those key individuals cannot continue their involvement, the projects may be interrupted or fail altogether (Li and Pang, 2022). Lastly, establishing stable cooperation networks among artists, community members, and public funders is necessary. These networks typically involve vertical negotiations and collaborations with authorities or community managers, often influenced by political or commercial decision-making frameworks (Eynaud et al., 2018). Furthermore, participatory art projects depend on continuous funding to facilitate the implementations and to exert its impact and value. However, securing this funding poses significant challenges, which will be explore in the following section.

#### 3.3.4 Resource and funding problems

a  Funding support.

Findings from three studies demonstrate that one of the significant challenges in participatory art projects is securing resources or funding (Crisman, 2022; Haedicke, 2016; Phillips and Tossa, 2017). These projects typically rely on diverse investments from local governments, private enterprises, and non-governmental organisations. The uneven distribution and flow of funds often hinder the sustainability and expansion of these projects. For example, Phillips and Tossa (2017) shared a project that secured funding from the Australia–Thailand Institute. This shows that many projects’ ongoing success and expansion depend heavily on external funding, which affects their ability to sustain and grow. Therefore, project organisers must actively explore additional funding channels and develop innovative financing strategies to ensure the lasting impact of participatory art projects.

Additionally, as Haedicke (2016) pointed out, these projects often need ongoing financial support, which depends on the backing of city officials and community leaders. Due to the inconsistency of funding, the uncertainty of project sustainability, creating challenges for non-profit organisations, individual artists, and grassroots community initiatives, especially in bottom-up efforts (Crisman, 2022). While this type of organisation and mobilisation can yield positive results and practical significance, funding challenges persist.

b  Risks arising from funding support.

Evidence from three studies supports this finding (Eynaud et al., 2018; Irwandi et al., 2023; Kelaher et al., 2014). Irwandi et al. (2023) observed that while participatory art projects can attract tourists and transform locations into thriving tourist destinations, thereby boosting the local economy, these projects often struggle to operate independently without external funding. Funding is usually only available for initial physical transformations. Relying on external funds can lead to difficulties in maintaining the projects once the funding runs out. Kelaher et al. (2014) noted that there may be tensions between the artistic objectives and the priorities of funding bodies. Eynaud et al. (2018) further pointed out that projects sometimes have to secure funding through competitive processes, which may push them to engage in politicised or commercialised activities. This method of fund allocation pressures projects to become formalised, exposing participatory art to the risks of politicisation and commercialisation, potentially reducing them to mere “tools for political and commercial interests.” This further validates the concerns raised at the beginning of this study regarding the application of participatory art.

## 4 Discussion

This study systematically delves into the values and challenges of participatory art in urban and community contexts, revealing how various stakeholders promote urban development and community building by fostering social empowerment, democratisation, identity formation, and cross-cultural as well as cross-class exchanges. Table 5 presents the thematic findings of this research.

**Table 5:** Values and challenges of participatory art.

Category	Theme	Key points
Values	Social empowerment and democratisation	Authentic interaction Democratic participation
	Multidimensional communication	Cross-cultural and cross-group communication Intergenerational, cross-disciplinary, and cross-class communication
	Enhanced community cohesion	Shared spaces Diversity in participation and expression
	Local cultural identity	Collective memory and cultural transmission Revitalising public spaces
	Educational promotion	Broad social education Knowledge sharing and skills education
	Economic benefits	Tourism and commerce Local economic growth
Challenges	Political and commercial antagonism	Politicisation tool Commercialisation tool Antagonism and conflict
	Issues of social participation and acceptance	Community and public acceptance Acceptance at the level of popular culture
	Sustainability issues	Form and structural limitations Limitations of impact Dependency limitations
	Resource and funding problems	Funding support Risks arising from funding support

### 4.1 Public participation and consensus on the project are crucial to implementation

The research indicates that participatory art effectively engages in local policies and community transformation by activating public spaces, fostering inclusive dialogues, and enhancing the representativeness of community members. These practices enhance community cohesion and help dismantle cultural and economic barriers, fostering understanding and cooperation among individuals from diverse backgrounds. Notably, marginalised groups can express their voices through these artistic forms, which is crucial for promoting social inclusivity and reducing cultural biases in society. For instance, the Painted Stories project in Vancouver enabled marginalised refugee participants to co-create a mural that integrated political demands with personal affirmations, resisting victim stereotypes and asserting narrative agency (Fobear, 2017). In Vilnius, the Street Mosaic Workshop involved marginalised elderly residents in creating ceramic artworks that enhanced visibility and local belonging (Lavrinec, 2014). These cases show how participatory art fosters both voice and social connection. As art theorist Grant Kester emphasises, the value of art lies not only in its visual representation but also in the dialogues and community engagement it inspires (Kester, 2004). This collaborative interaction can catalyse strong social and cultural connections, foster collective consciousness, and address urban and societal issues through artistic practices (Bourriaud, 2002). Therefore, extensive public participation and consensus are vital to the success of the project.

Further research indicates that participatory art significantly enhances economic potential, particularly by attracting tourism and stimulating local economic activities. These artistic endeavours beautify urban environments and also act as catalysts for local economic development. Furthermore, artistic expression strengthens local cultural identity, fostering pride among community members and a sense of belonging by exploring collective memories and personal histories. For example, in Java, Indonesia, previously marginalised local communities were transformed into vibrant tourist destinations through participatory mural projects that incorporated local culture, food, and festivals, successfully revitalising the local economy and strengthening place identity (Irwandi et al., 2023). Thus, the key to realising these positive values and effects of participatory art lies in the extensive public participation and broad consensus on the project. Participatory art typically involves a diverse group of participants, including the public, artists, government agencies, and other relevant organisations. Each of these stakeholders carries their own participation goals and plans. These diverse perspectives may lead to varying levels of community identification and involvement. Therefore, broad community engagement and consensus on project objectives are essential for the success of participatory art.

While many studies have highlighted the need for broad public consensus, however, it is important to note that participatory art does not always aim to promote inclusion or achieve consensus. As Kester (2004) emphasises, dialogical practices in participatory art often unfold within complex contexts of social inequality and do not presume immediate agreement. Meanwhile, Bishop (2004) argues that the political potential of participatory art lies in its ability to sustain tension, provoke disagreement, and critically engage with existing structures. Although their discussions emerge from broader debates in socially engaged art, these perspectives remain highly relevant to participatory art practices situated in urban and community contexts. From this perspective, participatory art helps cultivate social relationships while also creating spaces of contestation that expose and challenge entrenched power dynamics. Therefore, participatory art in urban and community development demonstrates significant political complexity: it may foster democratic participation and cultural empowerment, yet also serve as a critical space for addressing social tensions and antagonisms. Its political role should not be simplistically defined as inherently democratic or emancipatory, but rather understood in relation to the specific power dynamics and institutional settings that shape both the possibilities and the limits of participation and consensus.

### 4.2 Sustainability of participatory art is a key challenge

Despite many positive impacts of participatory art, one major concern is that it can be exploited for political and commercial purposes, raising issues of social acceptability. In some cases, art projects led by governments or stakeholders stray from their initial goal of promoting democracy, often prioritise political or commercial interests over community needs. For example, a participatory art project at Forest Houses, though carried out in the name of public engagement, was in fact implemented through a highly top-down structural approach (Sacco et al., 2019). According to Claire Bishop (2023), many participatory art projects fail to fulfil their democratisation promises due to the lack of true democratic practices. Therefore, project initiators must collaborate closely with the community to ensure that these art initiatives are not dominated by specific interests. It is also important to prevent residents from unwittingly becoming instruments in achieving individual political goals or economic gains. It is essential to guarantee that all participants have real power and influence in the decision-making process, and their artistic practices are in line with the community’s needs and goals (Bishop, 2023; Kester, 2004).

Additionally, sustainability presents a significant challenge for maintaining participatory art in community and urban settings. It must also continuously inspire collective action and shared motivation. Art theorist Nicolas Bourriaud emphasises that when temporary and contextual art projects cannot be sustained, the initial enthusiasm and action built on collaborative creation may gradually fade, leading to a further decline in community dynamism and participation (Bourriaud, 2002).

Sustainability directly or indirectly affects the acceptance and efficacy of participatory art within the community. It may provoke public introspection, questioning, or alignment with its values and culture. The Park Life project in Manchester serves as a positive example (Sachs Olsen, 2018). Benefiting from embedded facilitation and the gradual development of community ownership, it successfully sustained collective motivation, demonstrating how sustainability can help transform participatory initiatives into enduring forms of civic practice. It directly intervenes in public participation and actions, influencing the long-term impact of the project. Simultaneously, it is necessary to remain alert to potential political and commercial motives behind funding, as these factors can impact the long-term success of participatory art and the genuine benefits to the community. Thus, ensuring the sustainability of participatory art involves more than just tackling short-term challenges. It is crucial to achieve long-term success or impact from the art projects. This requires collaborative efforts from project initiators, funders, and community members, who must establish continuous support and active participation in art projects through a transparent and inclusive decision-making process.

## 5 Limitations

The limitations of this study stem from the lack of consideration for specific economic, political, and cultural contexts in the analysis of participatory art literature related to particular countries or regions. Variations in social structures, political systems, community governance models, and local traditions may directly or indirectly influence how participatory art is initiated, perceived, and sustained in different contexts. Therefore, future research could benefit from incorporating diverse geographical and cultural backgrounds and evaluating the actual impacts from the perspectives of citizens and communities, thereby enabling a more comprehensive examination of its long-term social, cultural, and political effects. Furthermore, this study did not strictly differentiate between primary and secondary stakeholders in participatory art. Future research could explore stakeholders’ roles to better assess the effectiveness of participatory art.

In addition, although this review applied a systematic search strategy following PRISMA and relied on the WoS and Scopus databases, the final sample still exhibits a clear geographic imbalance, with most studies originating from Europe and only limited representation from other world regions. This likely reflects a broader issue in the field, where academic publications discussing participatory art continue to be more commonly situated within Western contexts. However, socially engaged art is not solely rooted in West, but shaped by diverse cultural, political, and intellectual traditions (Castellano, 2021). Therefore, future research could incorporate other sources, such as local project reports, community archives, or non-indexed publications, including regional databases and grey literature, which may bring greater visibility to perspectives from regions that are currently underrepresented in indexed academic literature.

## 6 Conclusion

This study aimed to systematically explore the multifaceted role of participatory art in urban planning and community contexts. Through a thematic analysis of 20 published articles, this study sheds light on the multifaceted values and challenges associated with participatory art in urban and social development. Six key emerging themes were identified, namely social empowerment and democratisation; multidimensional communication; enhanced community cohesion; local cultural identity; educational promotion, and economic benefits. The findings indicate that broad public participation and consensus-building are essential in realising these values.

Additionally, four major challenges are identified, including political and commercial antagonism, issues of social participation and acceptance, sustainability issues, and resource and funding problems. Sustainability emerged as a significant challenge during the planning and implementation phases. The study emphasises that the quantitative differences between values and challenges do not imply that the advantages outweigh the challenges, nor do they suggest that the complexity or severity of the challenges is less than the benefits.

Moreover, this review highlights the complexity and diversity of participatory art, acknowledges that participatory art is a complex process influenced by various factors. While participatory art may not fully meet the expectations of all stakeholders, it offers diverse pathways to promote the development of cities and communities and significantly demonstrates the growing influence of art in societal matters. To ensure the viability of this form of participation, it is essential to understand its inherent complexities in advance and to take appropriate preventive and responsive measures. This involves balancing different interests and values, ensuring that the voices of all parties are heard and respected while also considering environmental sustainability and the effective use of resources.

## Data Availability

The original contributions presented in the study are included in the article/supplementary material, further inquiries can be directed to the corresponding author.
